# Body composition changes during 8 weeks of military training are not accurately captured by circumference-based assessments

**DOI:** 10.3389/fphys.2023.1183836

**Published:** 2023-06-07

**Authors:** Stephen A. Foulis, Karl E. Friedl, Barry A. Spiering, Leila A. Walker, Katelyn I. Guerriere, Vincent P. Pecorelli, David J. Zeppetelli, Marinaliz C. Reynoso, Kathryn M. Taylor, Julie M. Hughes

**Affiliations:** Military Performance Division, U.S. Army Research Institute of Environmental Medicine, Natick, MA, United States

**Keywords:** body fat, basic training, military personnel, dual-energy X-ray absorptiometry, anthropometry

## Abstract

In 1981, the US military adopted body fat standards to promote physical readiness and prevent obesity. Separate circumference-based equations were developed for women and men. Both predictive equations were known to underestimate %BF. However, it was not known how well these abdominal circumference-based methods tracked changes in %BF. This study examined the validity of the circumference-based %BF equations for assessing changes in %BF in young adult recruits during Army Basic Combat Training (BCT). Dual-energy X-ray absorptiometry (DXA) and circumference-based measures of %BF were obtained in women (*n* = 481) and men (*n* = 926) at the start (pre-BCT) and end (post-BCT) of 8 weeks of BCT. Repeated-measure ANOVAs were used to assess differences between DXA and circumference pre-BCT and for the change during BCT. Pre-BCT, circumferences underestimated %BF relative to DXA, with mean errors of −6.0% ± 4.4% for women and −6.0% ± 3.5% for men (both *p* < 0.01), and no difference between sexes was observed (*p* = 0.77). DXA detected a −4.0% ± 2.4% and −3.3% ± 2.8% change in %BF for women and men in response to BCT, respectively (both *p* < 0.01), whereas circumference estimates of %BF indicated a 0.0% ± 3.3% (*p* = 0.86) change in women and a −2.2% ± 3.3% (*p* < 0.01) change in men (sex difference by technique *p* < 0.01). In conclusion, circumference-based measures underestimated %BF at the start of BCT in both sexes as compared to DXA. Circumference measures underestimated changes in %BF during BCT in men and did not detect changes in women. These findings suggest that circumference-based %BF metrics may not be an appropriate tool to track changes in body composition during short duration training.

## Introduction

Body fat standards were mandated within the U.S. Army in the early 1980s to motivate fitness behaviors, prevent obesity, and ensure a physically ready force (U.S. Department, 1981b; U.S. Department, 1981a). Current circumference-based methodology for measuring body composition in the U.S. Army was developed by the Navy in 1984 (Hodgdon and Beckett, 1984; Hodgdon and Beckett, 1984; Hodgdon, 1992). These equations were known to underestimate %BF relative to criterion methods, such as underwater weighing, in the upper ranges of body fat and overestimate %BF at the lower end of body fat (Hodgdon, 1992; Hodgdon and Friedl, 1999; Van Loan et al., 2001). Thus, it was considered an acceptable error, providing additional protection from the measurement error to overfat soldiers. Soldiers over the limits become ineligible for awards, classes, and promotions until they meet the standards. They must be remeasured monthly in order to determine whether they are making progress toward their target body composition, and if they are not making progress, they can be discharged from service (U.S. Department of the Army, 2019a). The simplicity and scalability of this approach have allowed circumference-based equations to be used as a commonplace estimate of %BF in many areas of the general population (American College of Sports Medicine, 2014).

In more than 30 years since military body fat standards have been in place, the makeup of the U.S. Army has changed. In particular, all job roles, including combat arms and Ranger positions, have recently opened for women, resulting in a substantial increase in the number of women in the U.S. Army with a greater lean mass than previously noted (McClung et al., 2022). Furthermore, the population of the United States, and thus the recruiting pool for the military, has become increasingly overweight and less fit (Bornstein et al., 2019), placing a greater need on Basic Combat Training (BCT) to produce positive body composition changes to ensure a fit and ready army. In this population, the majority of women and men lose %BF due to the loss of fat mass and gain of lean mass during BCT despite minimal changes in the total body mass when using DXA (Foulis et al., 2021). However, whether these favorable changes in body composition are captured by circumference-based anthropometry is unclear, questioning not only the tests used to measure changes in response to BCT but also changes that may occur in soldiers placed on a training program in order to improve body composition to meet the standards. Thus, the purpose of the current analysis is to determine how accurately circumference measures reflect the change in percentage body fat for men and women compared to the change determined by DXA, during 8 weeks of BCT.

## Methods

### Subjects

A total of 1,407 trainees (481 women and 926 men) with complete pre- and post-training DXA and circumference data participated in this subset analysis of the parent ARIEM Reduction in Musculoskeletal Injury (ARMI) study (Hughes et al., 2019). Volunteers were trainees in seven different BCT classes at Fort Jackson from 2018 to 2019. All participants were briefed on methodology and risks of participation prior to signing an informed consent document approved by the U.S. Army Medical Research and Development Command Institutional Review Board. Participants were between the ages of 17 and 42. In compliance with the Department of Defense Instruction (DoDI), 3,216.02 trainees who are 17 years of age are considered adults while in the federal duty status and are allowed to consent without parent or guardian approval. All participants were healthy at the start of BCT and free of any musculoskeletal injury (MSKI) that would restrict participation in physical training.

### Procedures

Data collection occurred during weeks 1 (pre-BCT) and 9 (post-BCT). Participants reported to data collection in a fasted state prior to breakfast and having not participated in physical training (PT). Height (cm) and total body mass (TBM, kg) were collected using a stadiometer and calibrated scale, respectively, while in standardized PT uniforms (athletic shorts and t-shirt) and without shoes. BMI (kg∙m^-2^) was calculated from this information.

Body composition was assessed using body circumferential taping in accordance with the methodology described in AR 600–9 (U.S. Department of the Army, 2019a). Briefly, circumferences were measured with flexible tapes held flat on the skin with standardized tension at the neck, waist (defined as the narrowest part of the torso), and hips (defined as the widest part of the buttocks) for women and at the neck and abdomen (at the level of the umbilicus) for men. Percentage body fat was estimated using the following equations (measurements in cm):

#### Women

% body fat = 163.205 x log_10_((waist + hip − neck)/2.54) − 97.684 x log_10_(height/2.54) − 78.387.

#### Men

% body fat = 86.010 x log_10_((abdomen − neck)/2.54) − 70.041 x log_10_(height/2.54) + 36.76.

When possible, the same researchers measured the circumferences at both timepoints; however, due to the travel requirements and long duration of the study, this was not always logistically possible. The male and female equations are previously reported to have a standard error of the estimate of 3%–4% BF and were reproducible by trained observers within 1% BF (Hodgdon and Friedl, 1999).

Body composition (%BF, lean mass) was also assessed by DXA (Prodigy, GE Healthcare, Madison, WI) later in the day (following breakfast or lunch), and data analysis was completed using manufacturer’s supplied algorithms (Encore, version 11.40, Lunar Corp., Madison, WI). DXA determined mass corresponded to gravimetric measures with high reliability at pre- and post-BCT, both with a correlation coefficient of > 0.99 for women and men and slopes ranging from 1.00 to 1.01. The coefficient of variation for DXA fat mass (FM) and %BF measurements have been previously reported to be <2% (Mazess et al., 1990; Toombs et al., 2012).

### Statistical analyses

Statistical analyses were performed using SPSS for Windows Version 26 (IBM Corporation, Armonk, New York). Descriptive statistics were calculated and reported as mean ± standard deviation (SD) for each group. Body fat data are presented as percentage points and percentage point changes unless otherwise stated. A two-way (sex × technique) ANOVA with time as a repeated measure was used to determine if the error in the % change measurement differed by sex. Bland–Altman plots are presented in the Supplementary Figures to provide the mean bias and 95% limits of agreement. Univariate linear regressions were used to analyze the relationships among pre-BCT, post-BCT, and change (across the 8 weeks of BCT) for %BF by circumference and %BF by DXA. Paired *t*-tests were used to assess the differences in the individual circumference sites within a sex.

## Results

### Descriptive anthropometric data

Anthropometric and %BF data measured at pre- and post-BCT are summarized in Table 1.

**TABLE 1:** Descriptive data and %BF for women (*n* = 481) and men (*n* = 926) before and after BCT.

	Women		Men	
	Baseline	Post-BCT	Baseline	Post-BCT
Age *yrs*	20 ± 4		21 ± 4	
Height *cm*	161.8 ± 6.5		176.0 ± 7.4	
Fasted mass *kg*	62.4 ± 8.9	62.6 ± 8.0	77.5 ± 12.8	75.9 ± 10.3
BMI *kg∙m* ^*-2*^	23.8 ± 2.8	23.9 ± 2.4	25.0 ± 3.7	24.5 ± 2.9
Lean mass *kg*	41.1 ± 5.7	43.9 ± 5.6*	57.8 ± 7.6	59.5 ± 7.2*^,^#
Circumference %BF	26.1 ± 5.5	26.1 ± 4.5	16.7 ± 6.5	14.5 ± 4.8*^,^#
DXA %BF	32.1 ± 5.2	28.1 ± 4.3*	22.8 ± 6.3	19.4 ± 4.6*

Mean ± SD. *p < 0.05 for the main effect of time. #*p* < 0.05 for sex × time interaction.

### Comparison of pre- and post-BCT %BF by technique

At the start of BCT, circumference-based equations estimated DXA %BF, with *r* ^2^ = 0.44 and SEE = 4.12% for women and *r* ^2^ = 0.72 and SEE = 3.42% for men (*p* < 0.01 for both). Circumference %BF underestimated DXA %BF in women (−6.0% ± 4.4% BF) and men (−6.0% ± 3.5% BF) (*p* < 0.01 for both), with no difference between sexes (*p* = 0.77). Pre-BCT circumferences overestimated %BF by more than one percentage point in 5.0% of women and 1.3% of men, potentially having a negative impact on their career (Supplementary Figure S1).

Correlations between %BF by circumference and %BF by DXA were lower in post-BCT than in pre-BCT (*r* ^2^ = 0.36 and SEE = 3.56% for women and *r* ^2^ = 0.54 and SEE = 3.28% for men, *p* < 0.01 for both). The mean error of circumference %BF compared to DXA was −2.0% ± 3.9% for women and −4.9% ± 3.5% for men (*p* < 0.01 for both) (Supplementary Figure S2). %BF was underestimated more in men than women (*p* < 0.01). Circumferences overestimated %BF by more than one percentage point in 19.1% of women and 3.1% of men post-BCT.

### Comparison of %BF change by technique

**FIGURE 1:**
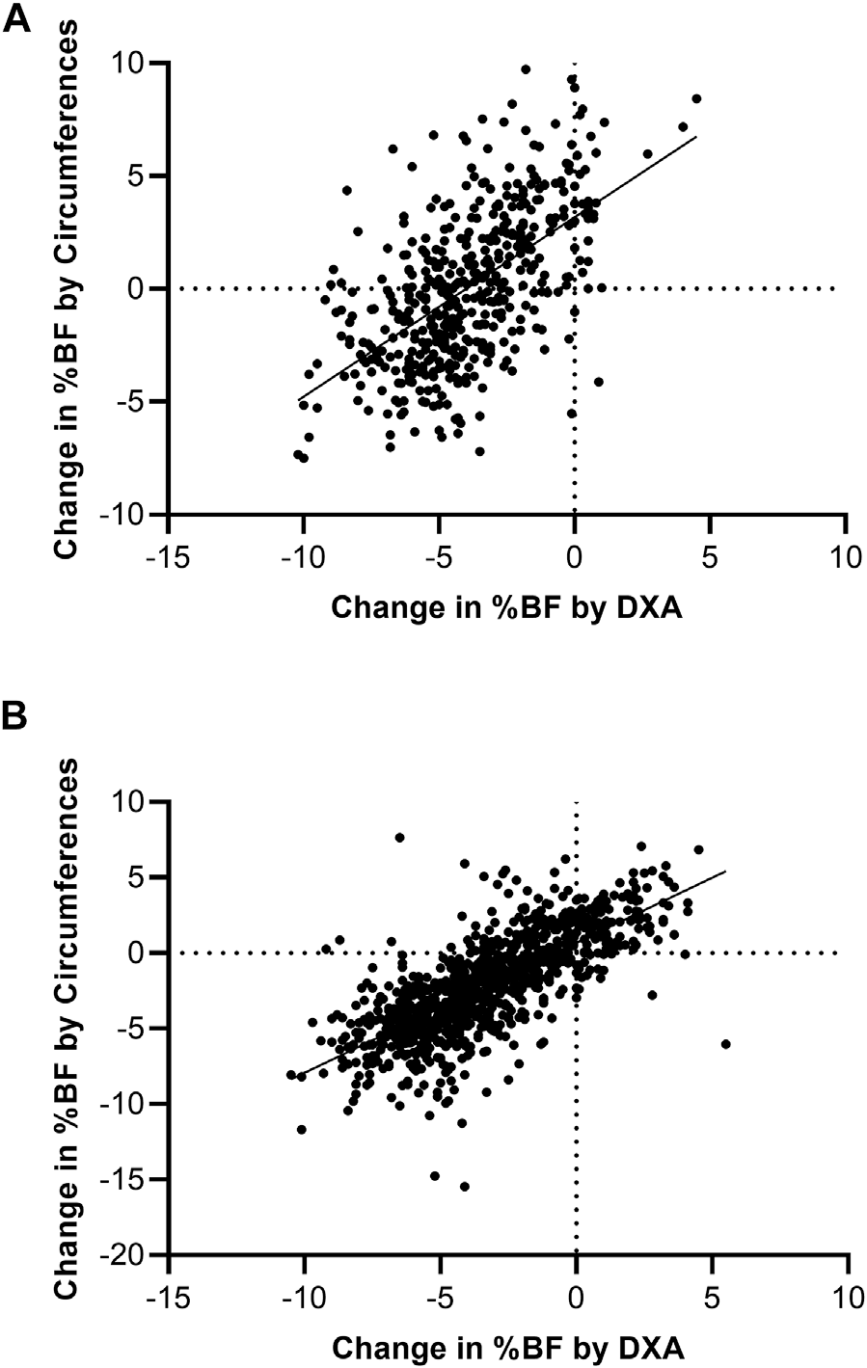
Change in circumference-based %BF plotted against change in DXA %BF for women **(A)** and men **(B)** during BCT. Dashed lines indicate where there is no difference between BF measures. In the upper graph, 93.3% of women reduced their DXA %BF during training, but this is not reflected in the circumferences. In the lower graph, men who reduced DXA %BF were better predicted by the male circumference-based %BF equation as having reduced %BF.

Most volunteers (93.3% of women and 86.1% of men) experienced decreases in %BF by DXA during 8 weeks of BCT. DXA detected a −4.0% ± 2.4% and −3.3% ± 2.8% change in %BF for women and men in response to BCT, respectively (both *p* < 0.01 for both), whereas circumference estimates of %BF indicated 0.0% ± 3.3% (*p* = 0.86) change in women and −2.2% ± 3.3% (*p* < 0.01) change in men (sex difference by the proposed technique *p* < 0.01). Changes in %BF as measured by DXA were better reflected in the circumference-based %BF in men than in women with an error of −1.1 ± 2.4% BF in men and −4.0 ± 2.8% BF in women (*p* < 0.01). Approximately 56% of women and 83% of men were correctly identified by circumferences as having a gain or reduction in BF of at least 1 percentage point as compared to DXA %BF (Figure 1). Additionally, 43.0% of women and 14.5% of men gained %BF as measured by circumferences but lost %BF when measured by DXA.

Changes in the individual circumference sites are shown in Table 2. Notably, all sites showed no significant changes in women (*p* ≥ 0.09); however, both sites decreased in men (*p* < 0.01).

**TABLE 2:** Individual circumference measurements before and after BCT.

	Women		Men	
	Baseline	Post-BCT	Baseline	Post-BCT
Neck *cm*	32.18 ± 1.66	32.26 ± 1.56	38.13 ± 2.15	37.66 ± 1.80*
Waist *cm*	71.31 ± 6.02	71.50 ± 5.13		
Hips *cm*	94.55 ± 6.52	95.35 ± 5.65		
Abdomen *cm*			85.65 ± 9.73	82.20 ± 7.00*

Mean ± SD. *p < 0.05 for the main effect of time.

## Discussion

Even though both men and women significantly lost %BF during BCT, the magnitude of this loss was not appreciated by circumference methods compared to DXA, particularly in women. In our study, we observed a 4.0% decline in %BF in women that was not captured by circumference measurements. These observations are in line with the results of a prior study of female trainees using DXA and skinfold anthropometric assessments of %BF (Friedl et al., 2001). Multiple skinfold formulas in that study failed to demonstrate changes in %BF despite the 2.3% decrease observed by DXA (Friedl et al., 2001). The conclusion from that study was that fat and muscle distribution changes in women in response to exercise were inadequately predicted from anthropometric measurements. Notably, that study did not include a male comparison group. For men, the circumference equation for %BF estimation highlighted the value of a single abdominal circumference in tracking male body composition, with the changes measured in this study by DXA closely associated with the changes in abdominal circumference. This was previously observed in a more extreme form in fit lean male soldiers who had an average of 10 cm reduction in the abdominal circumference during 8 weeks of Ranger school (Friedl et al., 1994). The gender differences in the change of abdominal circumference measures observed in this study suggest that patterns of female subcutaneous fat loss in response to short duration training may be more diverse than the changes in abdominal fat in men. Notably, the women in this study had greater gains in lean mass than the men and that may also have contributed to the differences observed in the circumference measurement error. Future studies should assess additional body regions in order to better understand these sex differences.

In contrast to the current observations, these same equations have been shown to significantly overestimate %BF in female soldiers who have succeeded in the Ranger course (McClung et al., 2022) as well as female Marines (Potter et al., 2022). In the study of Marines, the largest overestimates of %BF by circumferences were in the leanest individuals (Potter et al., 2022), and thus the underestimation of %BF by circumferences in our population may be due to the greater %BF of women at the beginning of training. As these findings suggest a bias of the testing against women, it is important for future studies to address ways of mitigating this bias in order to make sure women are not unfairly facing negative career consequences due to the body composition standards.

While in general population, excess %BF is of concern due to metabolic and cardiovascular disease, it represents an additional operational concern to the military. Numerous studies have linked high %BF and low lean mass to decreased physical performance (Pierce et al., 2017), MSKI risk (Jones et al., 2017; Nye et al., 2018), and discharge from BCT (Knapik et al., 2001). These studies have only used anthropometric measures, such as BMI and body circumferences, to determine body composition due to the convenience and feasibility of capturing these data in a large sample size (Friedl, 2004). Because of the significance of these studies for determining the health and wellbeing of soldiers, they should be followed up with studies using DXA.

It should be noted that there may be a bias in the selection of these participants that may limit its interpretation. Men and women attending BCT had to meet certain BMI and circumference %BF standards in order to be enlisted (U.S. Department of the Army, 2019b). Thus, individuals exceeding these limits are not included in these analyses, and therefore, these findings may not be generalizable to the entire US population in this age range. Hydration was also not controlled for. While trainees were allowed to consume water *ad libitum* and they are encouraged by drill sergeants to stay hydrated, hydration was not assessed. Furthermore, the same researchers were not always available to measure circumferences on the same participants at both timepoints, possibly introducing errors in addition to the inherent measurement error with circumference-based techniques. Due to the large sample size, adequate training of staff, and unsystematic assignment of researchers to administer circumference measurements, this was unlikely to have a significant effect on the outcomes. Any error introduced is likely comparable to real-world scenarios, where serial measurements may not be performed by the same individuals. Additional research is warranted to determine if these findings can be replicated when measured by the same individuals.

In summary, circumference-based measures underestimated %BF at the start of BCT in both sexes, as compared to DXA. Circumference measures underestimated changes in %BF during BCT in men and did not appropriately detect changes in women. These findings suggest that circumference-based %BF metrics may not be an appropriate tool to track changes in body composition during U.S. Army BCT.

## Data Availability

The raw data supporting the conclusion of this article will be made available by the authors, without undue reservation.
