# Supplementary material for: Body composition changes during 8 weeks of military training are not accurately captured by circumference-based assessments
Source: Front Physiol. 2023 Jun 7;14:1183836. doi: 10.3389/fphys.2023.1183836 (PMC10282178; doi:10.3389/fphys.2023.1183836)
Supplement: Supplementary file 1 [file Image1.pdf]

Body composition changes during eight weeks of military training are not accurately captured by circumference-based assessments

Stephen A. Foulis*, Karl E. Friedl, Barry A. Spiering, Leila A. Walker, Katelyn I. Guerriere, Vincent P. Pecorelli, David J. Zeppetelli, Marinaliz C. Reynoso, Kathryn M. Taylor, Julie M. Hughes

*Corresponding Author: stephen.a.foulis.civ@health.mil

SUPPLEMENTAL FIGURE LEGENDS

Supplemental Figure 1. Circumference-based %BF plotted against DXA %BF for women (A) and men (B) at the start of training. The line of identity is shown to highlight the underestimation of %BF by circumference-based %BF.

Supplemental Figure 2. Circumference-based %BF plotted against DXA %BF for women (A) and men (B) at the end of training. The line of identity is shown to highlight the underestimation of %BF by circumference-based %BF.

Supplemental Figure 3. Bland Altman Plots for women (left) and men (right) for the difference between DXA and circumference-based %BF at the start of training (top), difference between DXA and circumference-based %BF at the end of training (middle), and difference between DXA and circumference-based changes in %BF (bottom). Solid line represents the mean bias, and dotted lines indicate the 95% limits of agreement.

Supplemental Figure 1

A

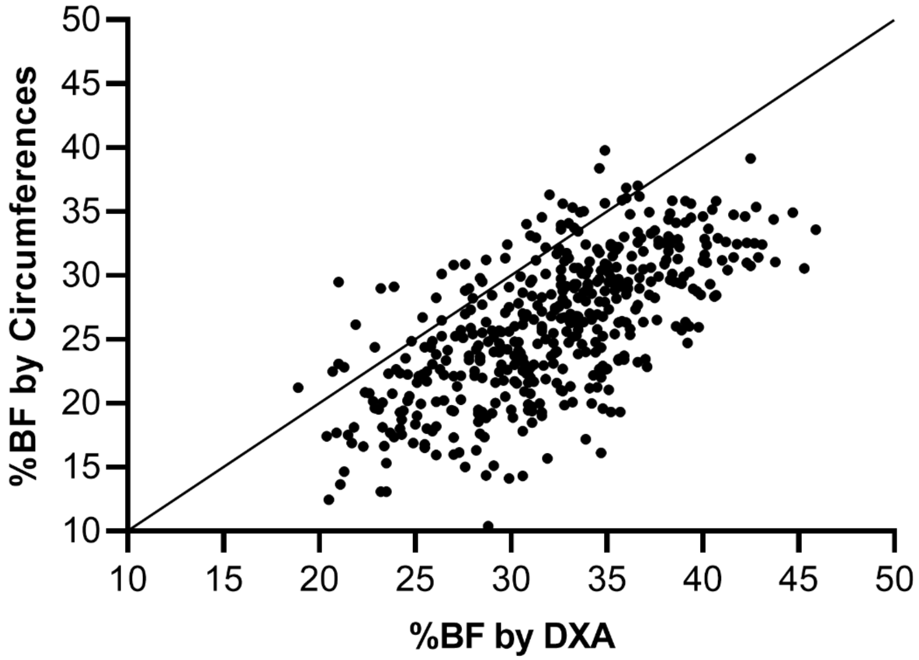

B

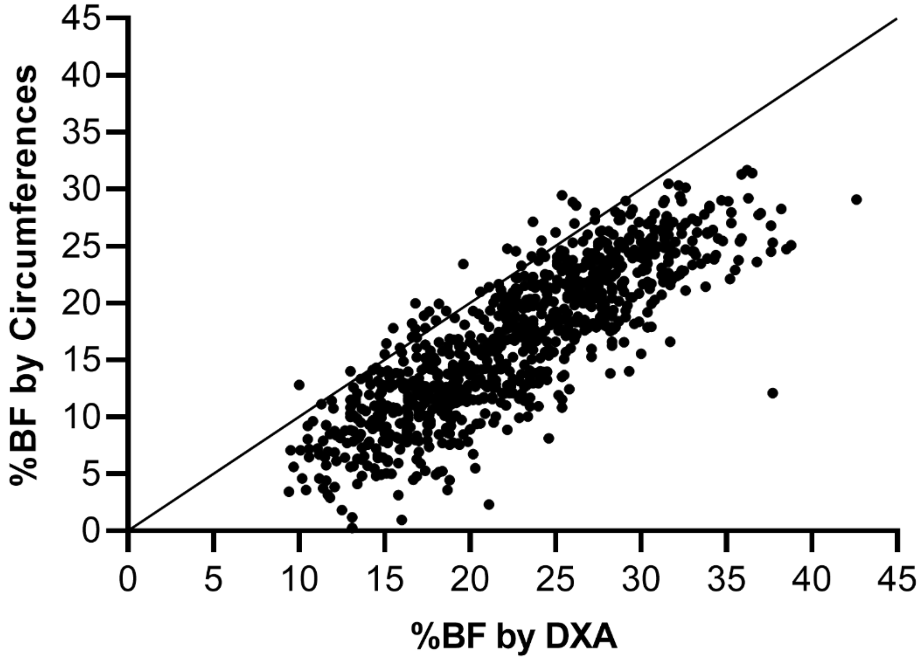

Supplemental Figure 2

A

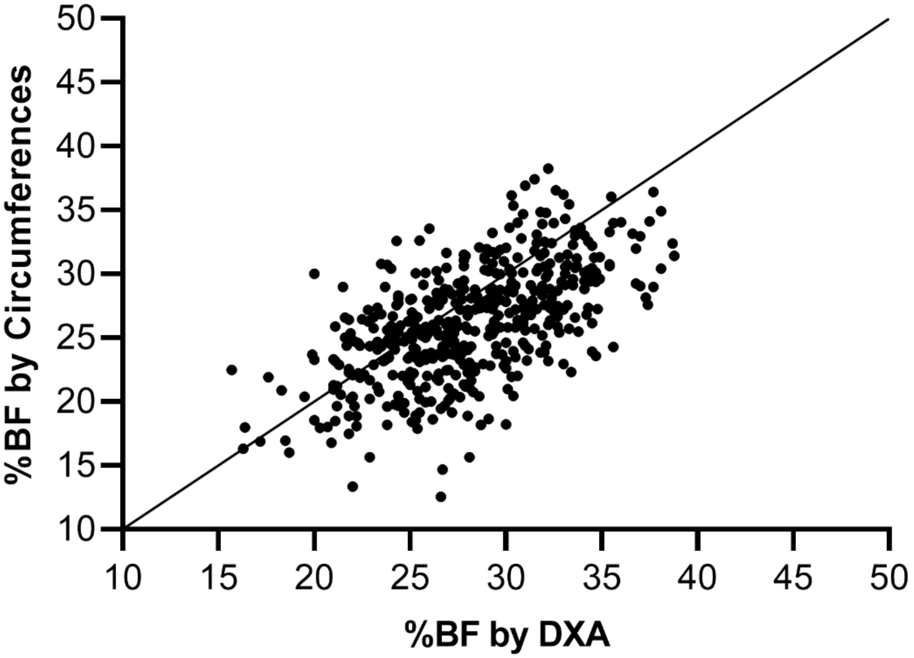

B

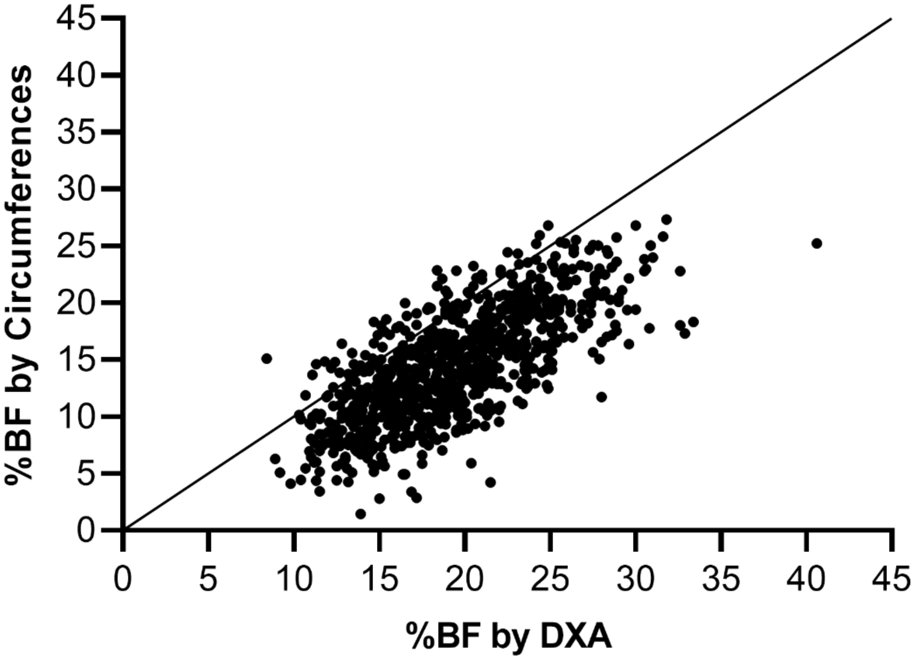

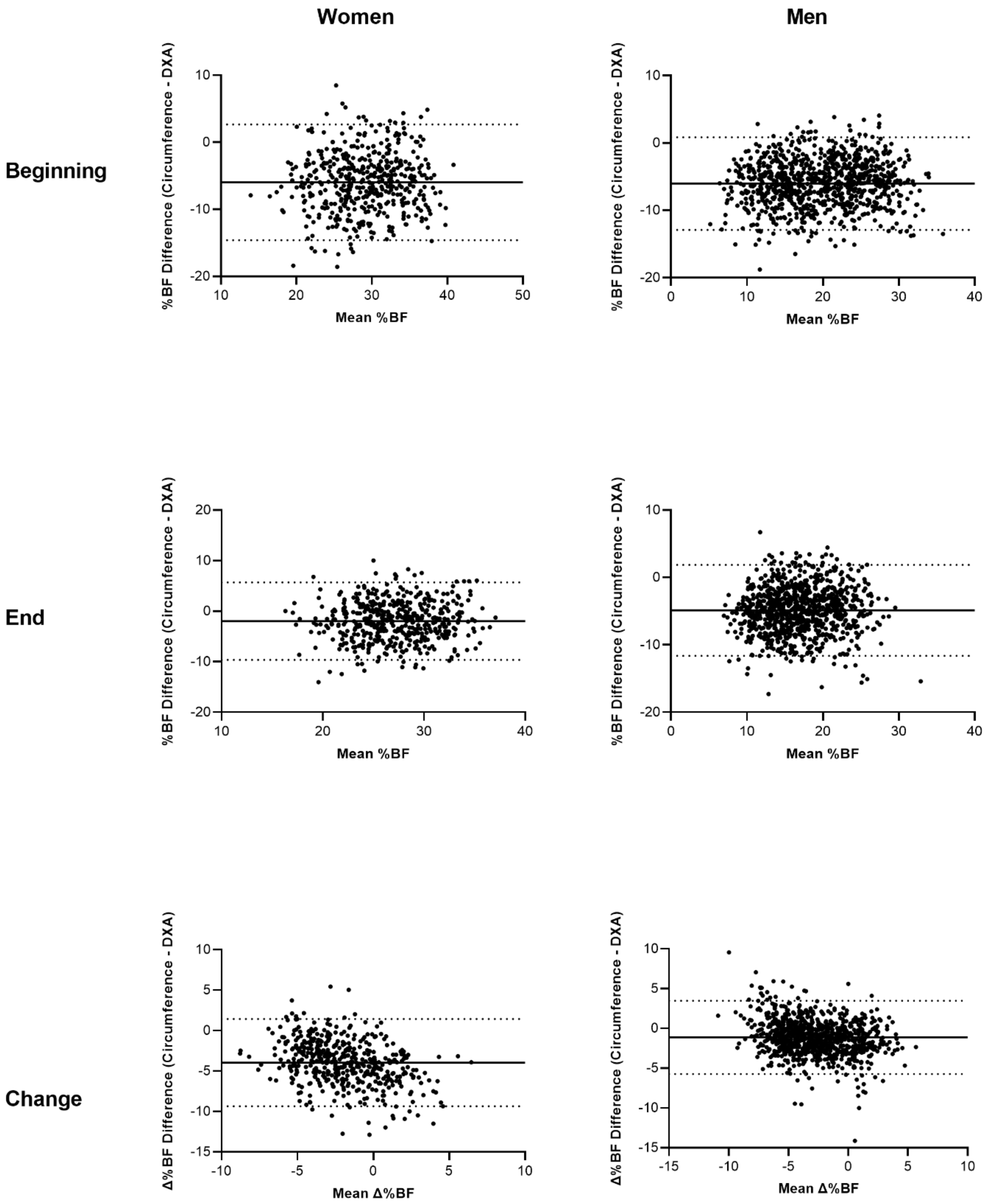
Women
Men
Beginning
End
Change
%BF Difference (Circumference - DXA)
Mean %BF
Δ%BF Difference (Circumference - DXA)
Mean Δ%BF
